# Detection and Characterization of Sindbis Virus Genotype IV in Mosquitoes From Slovenia

**DOI:** 10.1155/tbed/6371337

**Published:** 2026-01-20

**Authors:** Patricija Pozvek, Miša Korva, Samo Zakotnik, Tea Knapič, Katja Adam, Katarina Resman Rus, Gašper Grubelnik, Tomi Trilar, Vladimir Ivović, Tatjana Avšič-Županc, Nataša Knap

**Affiliations:** ^1^ Institute of Microbiology and Immunology, Faculty of Medicine, University of Ljubljana, Ljubljana, Slovenia, uni-lj.si; ^2^ Slovenian Museum of Natural History, Ljubljana, Slovenia; ^3^ Department of Biodiversity, Faculty of Mathematics, Natural Sciences and Information Technologies, University of Primorska, Koper, Slovenia, upr.si

**Keywords:** arboviruses, Genotype IV, mosquitoes, NGS, phylogenetic analysis, Sindbis virus

## Abstract

Mosquitoes play a crucial role as vectors of disease pathogens and are among the most socioeconomically important animals in the world. Medically important arboviruses include the Sindbis virus (SINV), which causes chills, skin rashes, and joint pain. Endemic in northern Europe, SINV has been increasingly detected in central Europe. The main objectives of this study were to monitor and screen mosquitoes for the presence of SINV. We included samples of mosquitoes collected throughout the years at different locations in Slovenia. The mosquitoes were first identified and then pooled according to species, sex, date of sampling, and location. Nucleic acid (NA) was isolated from these pools, and the target segment of the SINV genome was amplified using molecular methods. We performed detailed phylogenetic analyses of the SINV‐positive mosquito pools. From 2020 to 2024, we monitored mosquitoes at 226 locations in Slovenia and collected 112,001 samples, which were identified and grouped into 11,595 pools. Using real‐time reverse transcription polymerase chain reaction (RT‐PCR), we found SINV RNA in five pools of *Culex modestus* mosquitoes collected in two regions of northeastern Slovenia in August 2022 and in July, August, and September 2024. The SINV discovered in Slovenia (SINV‐SLO) was classified as Genotype IV. We designed a primer scheme for the whole‐genome amplification of SINV based on the alignment of the available SINV‐IV sequences and performed molecular characterization of the sequences. Our findings revealed that SINV‐SLO is closely related to the SINV strains identified in Russia, Azerbaijan, and China. In Europe, it is Genotype I that is most frequently detected and causes epidemics, whereas Genotype IV, which was detected in Slovenia, has not yet been associated with disease outbreaks.

## 1. Background

Sindbis virus (SINV) is an alphavirus transmitted by mosquitoes and birds with a wide geographical distribution across the Old World. Mosquitoes are the primary vectors of SINV, while birds are the main amplifying hosts [1, 2]. SINV is primarily transmitted by ornithophilic mosquitoes of the genus *Culex* but also by *Culiseta* and *Aedes* [3]. Despite its wide distribution, symptomatic human infections have only been reported in a few geographically restricted areas, such as northern Europe, and occasionally in South Africa [4], Australia [5], and China [6]. Phylogenetic analysis of the partial E2 gene has identified six SINV genotypes (I–VI). Genotype I (SINV‐I) has been found in Europe, Africa, and the Middle East, SINV‐II and SINV‐VI in Australia, SINV‐III in Southeast Asia, SINV‐IV in Asia and the Middle East, and SINV‐V (also known as Whataroa virus) was detected in New Zealand [7]. Very little is known about Genotype IV. Phylogenetic analyses show that Genotype IV includes strain KYZV LEIV‐65A, strain Stavropol, and Chinese strain XJ‐160 [8]. Strain KYZV was first detected in *Culex modestus* mosquitoes collected in the Kyzyl‐Agach game preserve on the shores of the Caspian Sea in southeastern Azerbaijan in a breeding colony of ardeid birds in 1969 [9]. A virtually identical strain XJ‐160 was detected in *Anopheles* sp. mosquitoes captured in a rice field at the Yili River in Xinjiang, China, in 1990 [10], with only 0.01% difference in nucleotides (nts) and amino acids (aas) between strains [11]. Although 19% of subjects in a Chinese seroprevalence study had positive antibody titers against XJ‐160, SINV‐IV has never been associated with human disease [8].

The aim of this surveillance project was to detect the virus and its possible circulation in Slovenia by analyzing the presence of SINV in mosquitoes.

## 2. Materials and Methods

### 2.1. Field Sampling and Preprocessing of Mosquitoes

From 2020 to 2024, we carried out monitoring of vectors and vector‐borne pathogens in Slovenia. Mosquito samples were collected from 226 locations across the country between April and October, with each location sampled once a month. The mosquitoes were sampled using three types of traps: BG‐Sentinel traps, CDC light traps, and Gravid traps. The BG‐Sentinel and CDC light traps were baited with dry ice, whereas, in the Gravid, mosquito trap water was used as an attractant. All traps were placed in natural areas near water sources or animals, such as cows, sheep, and hens. They were left active for approximately 24 h to capture both diurnal and nocturnal mosquito species. The captured mosquitoes were transported to the laboratory on dry ice and stored at −30°C for an average of 5 days, until morphological identification.

The mosquitoes were identified to species level and grouped into pools of up to 50 individuals according to species, sex, location, and date of sampling. Identification was based on the morphological key “MosKeyTool, an interactive identification key for mosquitoes of the Euro‐Mediterranean” [12]. Where morphological identification was unclear, species were determined by amplifying and sequencing of the cytochrome oxidase I (COI) gene, using the method introduced by Folmer et al. [13]. During the high abundance periods, the mosquitoes were only pooled by genera to preserve the integrity of samples; therefore, about 20% of mosquitoes were not identified to species.

The preparation of the homogenates and the pipetting of the samples for nucleic acid (NA) isolation were both carried out in biological safety cabinets. Mosquito samples were homogenized in 600 μL of RPMI‐1640 medium (SIGMA, United Kingdom) with a 5 mm stainless steel ball (Qiagen, Germany) using a TissueLyser homogenizer (Qiagen, Germany) for 5 min at a frequency of 30 cycles per second. Following homogenization, the samples were centrifuged for 5 min at 14,000 rpm in an Eppendorf MiniSpin centrifuge (Eppendorf AG, Germany), and the supernatant was transferred to a clean microcentrifuge tube.

### 2.2. SINV Molecular Detection

For NA isolation, 300 μL of the homogenized supernatant was used. NA was isolated using a commercial TANBead OptiPure Viral Nucleic Acid Extraction Kit with the automated TANBead MAELSTROM 9600 (TANBead, Taiwan). Following isolation, the NA and homogenates were stored at −80°C.

SINV was detected by real‐time reverse transcription polymerase chain reaction (RT‐PCR) as described by Jöst et al. [14]. Real‐time RT‐PCR was performed using a QuantStudio 7 system (Applied Biosystems, USA). Reactions were carried out in a total volume of 12.5 μL and contained 5 μL of RNA, 2.5 μL of TaqMan Fast Virus 1‐Step Master Mix (Applied Biosystems, Thermo Fisher Scientific, Grand Island, NY, USA), 0.5 μmol of each primer and 0.3 μmol of probe, and water. Cycling conditions were as follows: 50°C for 5 min, 95°C for 20 s, followed by 40 cycles of 95°C for 3 s and 60°C for 30 s.

### 2.3. Oligonucleotide Primer Pairs for Amplicon Amplification of SINV Genome

To enable rapid genotype determination and subsequent design of genotype‐specific primers for whole‐genome amplicon sequencing, an alphavirus‐generic RT‐PCR targeting a conserved region of the nsP4 gene was performed on the first samples positive by real‐time RT‐PCR [15]. The nt sequence was determined by Sanger sequencing with genetic analyzer SeqStudio 8 Flex Genetic Analyzer (Thermo Fisher Scientific, USA). The partial nsP4 sequences provided sufficient phylogenetic resolution to assign the detected viruses to the appropriate SINV genotype, thereby enabling the immediate design of genotype‐specific primers for subsequent whole‐genome amplicon sequencing. Primer pairs for the amplification of the complete SINV genome were designed using the PrimalScheme tool [16]. Aligment of all four available complete SINV Genotype IV genomes (GenBank Accession Numbers: AF103728.1, KF981618.1, MG679375.1, and NC_075007.1) served as the basis for primer design. The sequences were aligned with MAFFT (v. 7.490) [17, 18]. The designed primer pairs can be found in Supporting Information 1: Table S1. We designed an amplicon system for sequencing genome of SINV Genotype IV, from 58 to 11,442 nt with 37 pairs of primers.

### 2.4. PCR Amplification and Optimization of Primer Pools

The PCR amplicons were produced with PrimeScrip One Step RT‐PCR Kit Ver.2 (TaKaRa, Japan). Final primer pool concentration in reaction was 10 μM. The concentration of purified amplicons was measured using the Qubit dsDNA HS Assay Kit on Qubit 3.0 (both Thermo Scientific Inc., Waltham, MA, USA). To reduce the potential interaction between primers in the primer pools, we divided the original two pools into nine separate primer pools as indicated in Supporting Information 1: Table S1.

### 2.5. Sequencing of Amplicons

Amplicon NGS libraries were prepared with Native Barcoding Kit 96 V14 SQK‐NBD114.96 according to the product instructions. The concentration of NGS libraries was measured using the Qubit dsDNA HS Assay Kit on the Qubit 3.0 (Thermo Scientific Inc.). Amplicons were sequenced on GridION running MinKNOW (v.24.11.8) and MinKNOW Core (v. 6.2.6) using Flow Cell (R10.4.1) (Oxford Nanopore Technologies, United Kingdom). Reads were basecalled using super accurate‐basecalling (v.4.3.0, 400 bps) with Dorado (v.7.6.7).

### 2.6. Bioinformatic and Phylogenetic Analysis

We used FastQC [19] for the quality check of the raw data. We then trimmed the raw reads with fastp [20] and checked the efficiency of the trimming with FastQC [19]. Trimmed reads were mapped to the reference genome AF103728.1 using Minimap2 [21] with default settings. The mapped reads were further processed using Samtools [22]; they were exported as a bam file that was sorted and mate‐flagged, duplicate alignments were marked, and the file was indexed. Primer sequences were trimmed using iVar trim [23]. The depth of coverage was calculated using the Samtools depth function. The consensus sequence was generated using Samtools consensus with the settings “‐‐min‐MQ 10 ‐C 10 ‐d 10”. Variants were called with iVar, and the effect of found alleles was predicted with SnpEFF [24].

The five newly generated SINV sequences from Slovenia and 69 whole‐genome SINV sequences obtained from the NCBI database were aligned with MAFFT (v. 7.526) [17, 18]. The resulting alignment was used to generate the phylogenetic tree with IQ‐TREE v 3.0.0 and the following settings “‐m MFP ‐bb 1000 ‐bnni ‐nt AUTO ‐alrt 1000‐abayes” [25–27]. The best fitting model was GTR + F + I + G4. The phylogenetic tree was created with the program TVBOT [28].

For the molecular clock analysis, a subset of the SINV sequences was prepared based on temporal structure and root‐peak regression analysis using TempEst v.1.5.3 [29]. Bayesian Markov chain Monte Carlo (MCMC) analysis was performed using Beast v.2.7 [30]. We performed 60 million Bayesian MCMC generations with strict clock and coalescent Bayesian skyline model and sampling every 1000 generations. We assessed convergence using Tracer v.1.7.2 [31]. Maximum clade credibility (MCC) tree was created with TreeAnnotator using 10% burn‐in. Posterior probabilities were calculated for each branch, nt substitution rates, and divergence times, with error expressed as 95% highest probability density (95% HPD). The MMC tree was created using TVBOT [28].

## 3. Results

### 3.1. Mosquito Sampling and Virus Genome Detection

Between 2020 and 2024, a total of 112,001 mosquitoes were caught and grouped into 11,595 pools. The mosquitoes were categorized according to species, sex, date of sampling, and location. The captured mosquitoes belonged to the genera *Aedes* (70%), *Culex* (23%), *Anopheles* (4%), *Culiseta* (1%), and *Coquillettidia* (2%).

**Figure 1:**
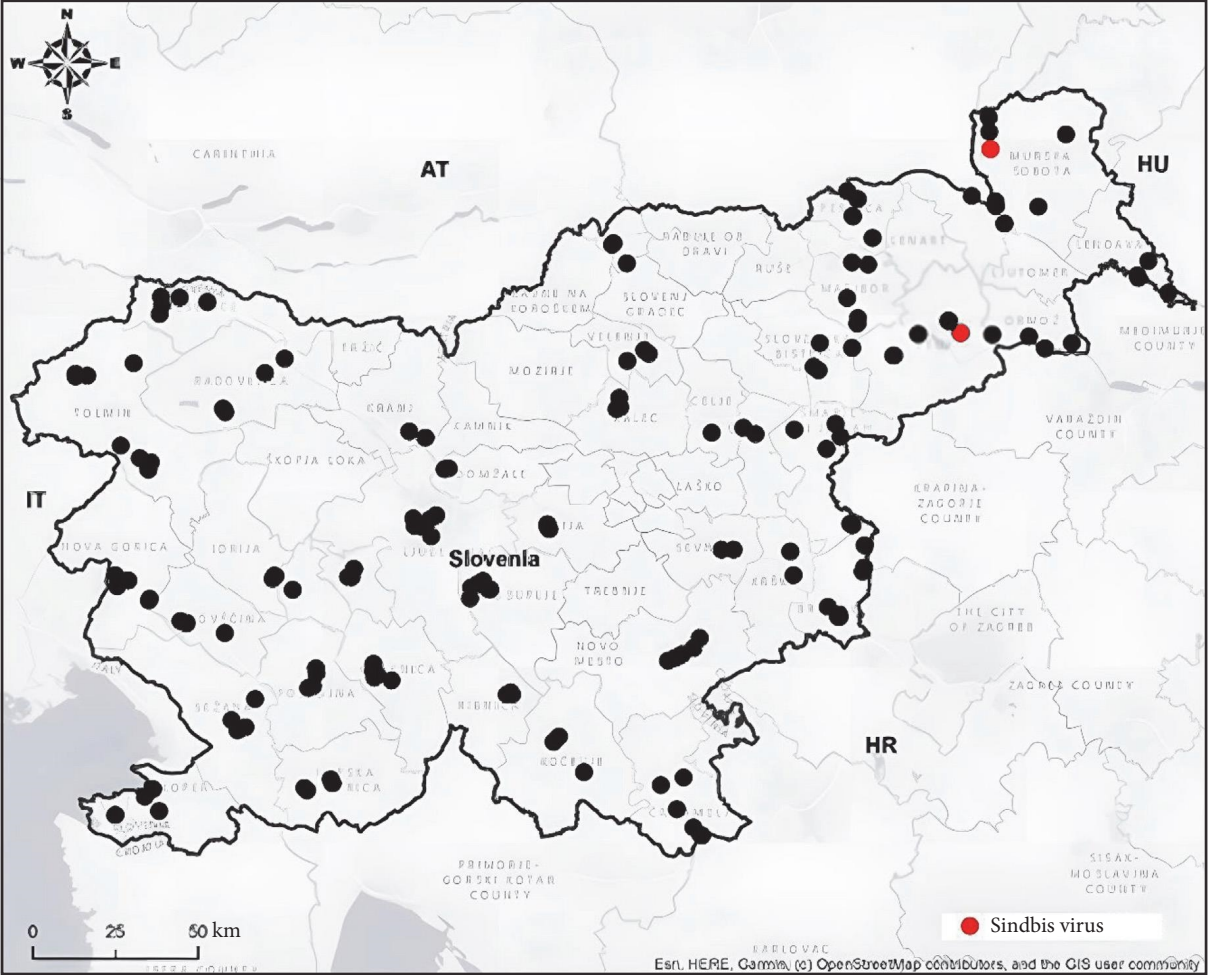
Locations of the mosquito sampling in Slovenia. The red dots show the locations where SINV‐positive mosquitoes were sampled. Upper dot represents Pertoča‐Ledavsko jezero, where SINV was detected in mosquito pools in August 2022 and 2024. Bottom dot represents Ptuj‐Podvinci, where SINV was detected in mosquito pools in July and September 2024.

SINV was detected by real‐time RT‐PCR in five pools of mosquitoes (Ko 2013/22 with Ct value 27.9, Ko 2128/22 with Ct value 31.0, Ko 876/24 with Ct value 33.3, Ko 1186/24 with Ct value 29.7, and Ko 1390/24 with Ct value 28.8). Each pool consisted of female *C. modestus*, which were collected using CDC traps in the Pomurska region in August 2022 and 2024 and in the Podravska region in July and September 2024 (Figure 1). The majority of *C. modestus* mosquitoes were captured in the northeastern part of Slovenia, and in the two regions, they were more abundant than the *Culex pipiens* species.

From the obtained data, we were able to calculate the minimum infection rate (MIR) using Program PooledInfRate Version 3.0 (a Microsoft Excel Add‐In, developed by Brad Biggerstaff; CDC, Fort Collins, CO). The overall MIR per 1000 mosquitoes across all captured specimens was 0.31 in the Pomurska region in 2022 and 0.10 in 2024, while it reached 1.91 in the Podravska region in 2024.

Among *Culex* mosquitoes specifically, the highest MIR/1000 was recorded in the Pomurska region in 2022, at nearly 4.5. In 2024, values were substantially lower: the MIR/1000 in Pomurska dropped almost fivefold, and in Podravska it was more than 50% lower than the peak observed in Pomurska in 2022. The exact calculated values are shown in Table 1.

**Table 1:** MIR in *Culex* mosquitoes in regions that we detected SINV.

Year of sampling	Region	Number of tested pools	Number of positive pools	Number of individuals	MIR/1000 mosquitoes
2022	Pomurska	72	2	477	4.48

2024	Pomurska	96	1	1264	0.79
	Podravska	116	2	1070	1.91

### 3.2. Development of an Amplicon System for SINV Genotype IV Genome Sequencing and Molecular Characterization

The sequences obtained were compared with the most closely related sequences in the BLAST NT database. Based on the matches found in the BLAST NT database, sequences were most closely related to SINVs classified as Genotype IV, specifically isolates XJ‐160, Kyzylagach LEIV‐65A, Stavropol, and Volgograd 673/19.

We successfully generated almost complete genomes from all five SINV positive samples directly from the mosquito pool. Sequences have an average length of 11,382 base pairs and 48% GC content. Variant analysis has revealed that genomes contain on average 94 major synonymous variants (allele frequency above 0.5) and 35 major missense mutations (allele frequency above 0.5; Table 2) based on the reference genome AF103728.1. Genome sequence AF103728.1—strain XJ‐160, Genotype IV, isolated in China from *Anopheles* mosquitoes was closest match in NCBI nt database based on blast search and was chosen as reference for mapping reads and calling variants. Average sequencing depth was 94.

**Table 2:** Details of sequencing of SINV positive mosquito pools, where NCBI accession numbers for each mosquito pool are visible, as well as sequence lengths, GC content, number of mapped reads, and major synonymous and missense variants for each sequence.

Mosquito pool	NCBI accession number	Length (bp)	GC content (%)	Number of mapped reads	Major synonymous variant	Major missense variant
Ko 2013/22	PP994673.2	11,442	48.5	226,626	103	34
Ko 2128/22	PV890983.1	11,326	48.5	218,938	103	34
Ko 876/24	PV890984.1	11,446	48.5	197,582	81	34
Ko 1186/24	PV890985.1	11,252	48.3	241,435	96	36
Ko 1390/24	PV890986.1	11,446	48.5	264,043	87	37

**Figure 2:**
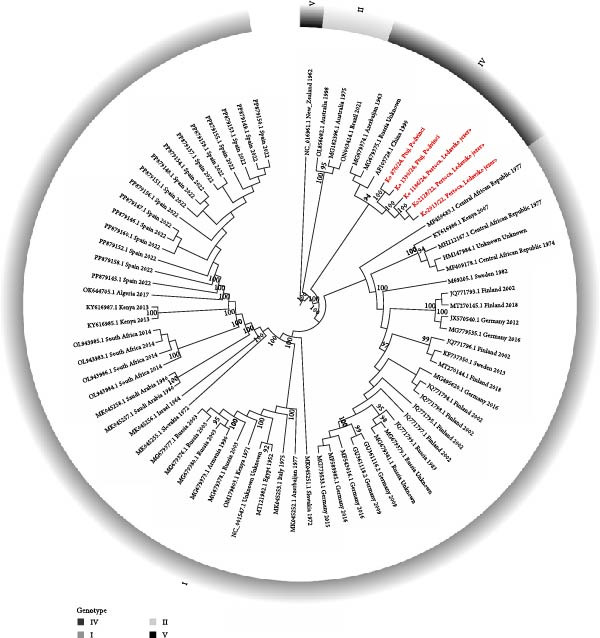
Phylogenetic analysis of selected SINV genomes from the NCBI (black) and new SINV genomes from Slovenia (colored red). All five Slovenian SINV genomes belong to Genotype IV. Ultrafast bootstrap values of 90 or higher are indicated on the branches of the tree. The SINV sequence NC_016961—Whataroa from New Zealand (SINV Genotype V) was used as the root. The GTR + F + I + G4 model was used for the tree reconstruction.

The five Slovenian SINV genomes represent a monophyletic cluster in Genotype IV, which differ by less than 2% at the nt level from other Genotype IV SINV genomes (Figure 2). By comparison, there is less than 80% nt identity between the new SINV genomes and those from Genotype I and even less (70%) with those from Genotypes II and V. Similar trends are observed at the protein level, with 99% aa identity within Genotype IV (for both structural and nonstructural proteins) and lower identity rates of 94%–96% for Genotype I, 91%–93% for Genotype II, and only 78%–89% for Genotype V.

### 3.3. Molecular Characterization of SINV Sequences

Comparing Slovenian SINV sequences with the NCBI reference SINV genome (NC_001547; Genotype I, host and country unknown) revealed 2149 nt differences (18%). The differences were as follows: nsP1, 233 nt; nsP2, 461 nt; nsP3, 382 nt; nsP4, 356 nt; capsid C, 127 nt; E3, 37 nt; E2, 277 nt; 6 K, 33 nt; E1, 243 nt. But a comparison with the closest sequence from XJ‐160 (AF103728.1) revealed only 233 nt difference. Higher nt divergence was observed in nonstructural proteins (nsP1, 23 nt; nsP2, 55 nt; nsP3, 43 nt; nsP4, 38nt), while in structural proteins only few mismatched were detected. The Slovenian SINV sequences had only 33 aa changes compared to the NCBI reference sequence and 52 in comparison to XJ‐160. The aas differences between Slovenian SINV sequences, reference strain (NC_001547), and XJ‐160 strain (AF103728.1) can be found in Supporting Information 2: Table S2.

### 3.4. Molecular Clock

**Figure 3:**
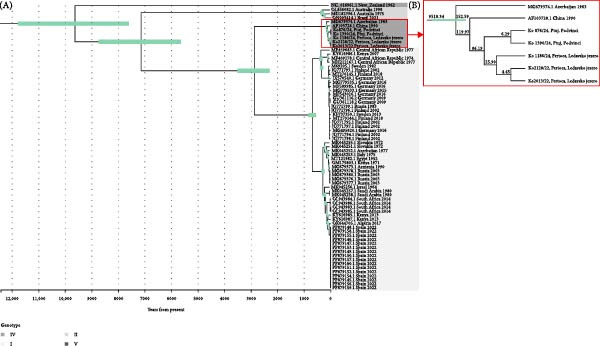
(A) Maximum clade credibility tree for 71 SINV sequences. The 95% HPD (highest posterior density) intervals are marked in green. Posterior probabilities (0.9–1) are indicated on the tree. Genotypes are labeled in different colors. The time axis is annotated in years before present. (B) Based on the available Genotype IV sequences, we constructed a MRCA (most recent common ancestor) tree.

The evolutionary rate of SINV Genotype IV was found to be 6.15 × 10^−5^ substitutions per site per year (median = 6.15 × 10^−5^; 95% HPD : 4.9 × 10^−5^ – 7.4 × 10^−5^). The most recent common ancestor (tMRCA) of the Slovenian SINV sequences dates back 85 years (95% HPD : 67.0–107.7 years). The closest strain, XJ‐160, was sampled in China in 1990; however, our molecular clock model estimated the tMRCA of Slovenian strains and XJ‐160 to be 121 years (95% HPD : 96.2–148.7 years; Figure 3).

The SINV sequence from Slovenia is most similar to a SINV sequence from China, which was isolated in 1990. However, the two populations appear to have diverged more than a 100 years ago.

## 4. Discussion

Over 4 years of monitoring vector‐borne pathogens in Slovenia, we captured 112,001 mosquitoes at 226 locations. Out of 11,454 mosquito pools, SINV RNA was only detected in five of them, all of which were collected in the northeastern part of Slovenia, near Lake Ledava and in the Podvinci nature reserve. SINV was detected in *C. modestus* mosquitoes, a species widely distributed in the temperate regions of Europe, Asia, and North Africa. SINV has previously been detected in *C. modestus* during field surveillance in the Czech Republic, Spain, and Romania; however, no experimental studies on the vector competence of this species for SINV have been conducted to date [8, 32–34]. These mosquitoes are most abundant in the summer and prefer to breed in permanent vegetative habitats such as rice fields and swamps. *C. modestus* mosquitoes feed on many bird species and are also anthropophilic, making them important vectors of emerging viruses [34]. The ponds in Podvinci are an important nesting area for many bird species, as is Lake Ledava, which is also a migratory flyway, which likely plays a role in the introduction and/or establishment of SINV.

The transmission of an infection from a natural reservoir to humans is influenced by several factors, including various environmental conditions and human exposure. The latter is influenced by the level of human activity in affected areas as well as the abundance and infection rate of the vectors. In 2022, the MIR in *Culex* mosquitoes in Pomurska region was 4.48 per 1000. In 2024, the MIR in *Culex* mosquitoes in Pomurska region was 0.79 and in Podravska region 1.91 per 1000. By way of comparison, Germany has repeatedly detected SINV in mosquitoes, yet there have never been any reported cases of human Sindbis fever. The recorded MIR was ~0.9 per 1000. In contrast, the MIR in the endemic area of Sweden is substantially higher, reaching 16.7 per 1000, nearly four times the observed rate in Slovenia in 2022 (4.48 per 1000) [35, 36]. Such difference in the MIR could be one explanation why no SINV infections in humans have been detected in Slovenia. Another possible explanation for the absence of detected human SINV infections in Slovenia could be the specific genotype of the virus detected in the country. Phylogenetic analysis of the SINV discovered in Slovenia (SINV‐SLO) sequences, obtained from a *C. modestus* mosquitoes, revealed that it is most similar to the XJ‐160, Kyzylagach‐LEIV65A, and Stavropol isolates, all of which are assigned to Genotype IV. None of the viruses detected in this group have been associated with human diseases. While most European SINV isolates, which cause disease in humans, belong to Genotype I, SINV‐SLO differed from it by an average of 17.7% at the nt level. The SINV sequences of Genotype II and Genotype V, which were detected in vectors in Australia and New Zealand, differed even more. The SINV isolate KYZV, which also belongs to Genotype IV and differs from SINV‐SLO by 1.5% at the nt level, was detected in mosquitoes of the same species in Azerbaijan in 1969 and in the Czech Republic in 2014 [8, 10]. Based on the sequence data, it is likely that SINV‐SLO was introduced to Slovenia by birds from the northeast as the sites are flyway points and nesting areas for many birds from northern and eastern Europe [37, 38].

Previous studies of the introduction and establishment of SINV‐I suggest that it was introduced to northern Europe from Central Africa only once, in the 1920s [7]. A recent study also described a new introduction and establishment of the SINV in Southern Spain. tMRCA of the Spanish sequences dated the introduction back to 4.66 years [33]. Since we detected and succesfully sequenced SINV Genotype IV in two different locations in two nonconsecutive years, we aimed to study the time to tMRCA. Therefore, we calculated the evolutionary rate of SINV Genotype IV to be 6.15 × 10^−5^ substitutions per site per year, which is comparable to the evolutionary rate of SINV Genotype I [33]. According to the calculated molecular clock, we would expect to see 1.4 nt substitutions in the virus sequences of the samples collected from the same location (Lake Ledava) in 2022 and 2024, provided that the virus overwintered in the local bird population. However, the sequences from Lake Ledava in 2022 and 2024 differed in 49 nts. The same number of differences was observed in SINV, detected at two sites in the northeastern part of Slovenia (Podvinci and Lake Ledava) within the same year. Using the molecular clock, we calculated that the estimated time to tMRCA between the SINV sequences sampled at the same site in 2022 and 2024 was 25.9 years. Based on the tMRCA, we hypothesize that there have been at least three separate introductions of SINV Genotype IV in Slovenia, rather than local overwintering. However, more SINV Genotype IV sequences would be needed to confirm the overwintering hypothesis. Our results show significantly greater differences between our sequences.

In order to gain a better understanding of the circulation of SINV at ponds in Podvinci and Lake Ledava, it would be beneficial to increase the intensity of mosquito sampling both during the period when SINV was first detected in mosquitoes and in the months before and after. Future research could include the determination of specific antibodies in people living in or visiting the area with positive mosquito pools to assess potential human exposure to SINV, as well as the determination of specific antibodies in birds in the affected area, as they are a reservoir for SINV.

## 5. Conclusion

Our study is the first to confirm the presence of SINV in vectors in Slovenia. We developed an amplicon sequencing scheme and found that SINV‐SLO belongs to Genotype IV. This genotype of SINV is more prevalent in Eastern Europe, Russia, and Asia. Our findings emphasize the importance of vector monitoring and surveillance of emerging pathogens in Europe.

## Conflicts of Interest

The authors declare no conflicts of interest.

## Funding

The study was supported by the Slovenian Research Agency (Grant Number V3‐2313: Monitoring of vector‐borne pathogens in vectors in Slovenia and V3‐1903: Establishment of monitoring of vectors and vector‐borne diseases in Slovenia, P3‐0083: Host–parasite relationship) and the Ministry of Health and Ministry of the Environment and Spatial Planning.

## Supporting Information

Additional supporting information can be found online in the Supporting Information section.

## Supporting information


**Supporting Information 1** Table S1. The designed primer pairs for sequencing genome of SINV Genotype IV, from 58 to 11,442 nt with 37 pairs of primers.

**Supporting Information 2** Table S2 shows amino acids differences between Slovenian SINV sequences, reference strain (NC_001547) and XJ‐160 strain (AF103728.1).

## Data Availability

All generated viral genome sequence acquired in this study was deposited with NCBI under Accession Numbers PP994673.2, PV890983.1, PV890984.1, PV890985.1, and PV890986.1. Additional data are available upon request from the corresponding author.
